# Being close to an election does not make health more politically relevant: more experimental evidence during a global pandemic

**DOI:** 10.1136/bmjgh-2020-004296

**Published:** 2021-01-06

**Authors:** Arnab Acharya, John Gerring, Aaron Reeves

**Affiliations:** 1 Independent Researcher, Washington, UK; 2 Department of Government, University of Texas System, Austin, Texas, USA; 3 Department of Social Policy and Intervention, Oxford University, Oxford, Oxfordshire, UK

**Keywords:** health policies and all other topics, public health

**Summary box**

- At the onset of COVID-19, experimental surveys, conducted in India, the UK and the US, showed voters are unlikely to punish or reward politicians for their success or failure in managing the pandemic.
- Here we report that a follow up survey conducted only in the US three weeks before the national election showed results similar to those from the older survey.
- Support for the incumbent remains the same across treatments while all respondents are more likely to blame the government for allowing the virus to spread.
- Although unable to conclude that the pandemic has had no influence on electoral outcomes, our results do raise questions about whether and how political institutions might contribute toward improving health.

Our paper ‘Is health politically irrelevant?’,^1^ published in the BMJ’s recent series on ‘Democracy and Health’, reports results from a survey experiment conducted in April–May of 2020 in the USA, the UK and India. We found that exposing research subjects to statements about the expected economic and health impact of the pandemic had no discernible impact on whether they blamed the government for the spread of the virus or were more/less likely to support the incumbent in a (hypothetical) upcoming election. This result suggests that politicians are unlikely to be electorally punished or rewarded for their response to the pandemic.

However, there are several alternate explanations. First, because the surveys did not coincide with national elections in any of the three countries one may doubt whether responses to a hypothetical election reflect reactions during an actual election. Second, the pandemic was still in its early stages when the surveys were conducted and citizens might not have fully appreciated its gravity, or its longevity. Third, the treatments may not have been sufficiently pointed. We merely provided information about the impact of the pandemic, leaving respondents to decide whether politicians were in any way culpable.

Any or all of these factors might account for the null results obtained in our survey experiment. It therefore seemed advisable to replicate the survey at a later date in at least one country. We chose the USA as a national election was imminent. Accordingly, the follow-up survey was launched in mid-October 2020, finishing just a few weeks before the November election.

The second survey was designed as a replication of the first, drawing on the same M-Turk recruitment platform (which results in a small number of repeat respondents) and the same survey design—with two notable exceptions. First, treatments are altered to reflect current predictions about the likely economic and health effects of the virus. Second, a new, stronger treatment is introduced as a third treatment arm:

As you are probably aware, the Coronavirus disease (Covid-19) has spread around the world. Its impact has been especially severe in the United States, which has one of the highest infection rates of any country in the world. An estimated 250 000 people have already died from causes associated with the virus. Some blame this catastrophe on Donald Trump and the Republican Party. According to one estimate, approximately 50% of American deaths (roughly 125 000 deaths) would not have occurred if Trump had publicly acknowledged the severity of Covid-19 and followed expert advice on how to manage the shutdown of schools and businesses, social distancing, and the distribution of masks.

The specific language employed above is based on findings reported in a major study.^2^ Instead of merely reporting the possible impacts of the virus (as our other treatment arms do), this new arm locates blame for the pandemic squarely on the incumbent. As such, we have gone about as far as we reasonably could—without engaging in controversy—to connect public health to the actions of specific politicians.

As it happens, the results of this follow-up experiment do not deviate appreciably from those reported in the initial study. To be sure, respondents across the board (regardless of treatment condition) are now more likely to hold politicians accountable for the course of the pandemic, as shown in figure 1. However, this does not translate into diminished support for the incumbent, Donald Trump, as shown in figure 2. In other words, people recognise that the American government has made mistakes in its handling of the pandemic, but these mistakes are apparently not serious enough to affect support for the President.

**Figure 1:**
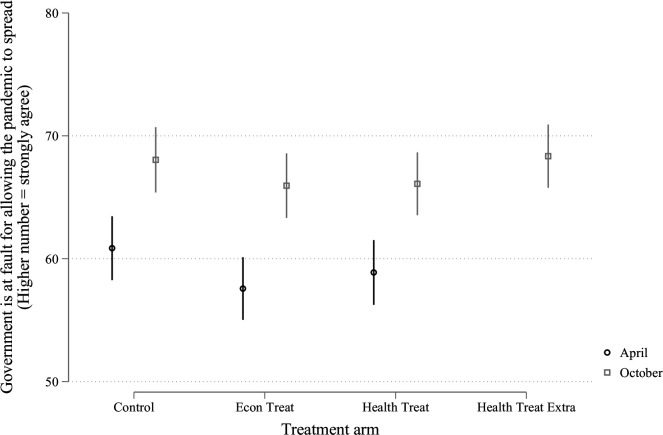
Government at fault. Figure contains data from the USA collected in April and in October. The April data were discussed in our earlier paper.^1^ Vertical lines are 95% CIs.

**Figure 2:**
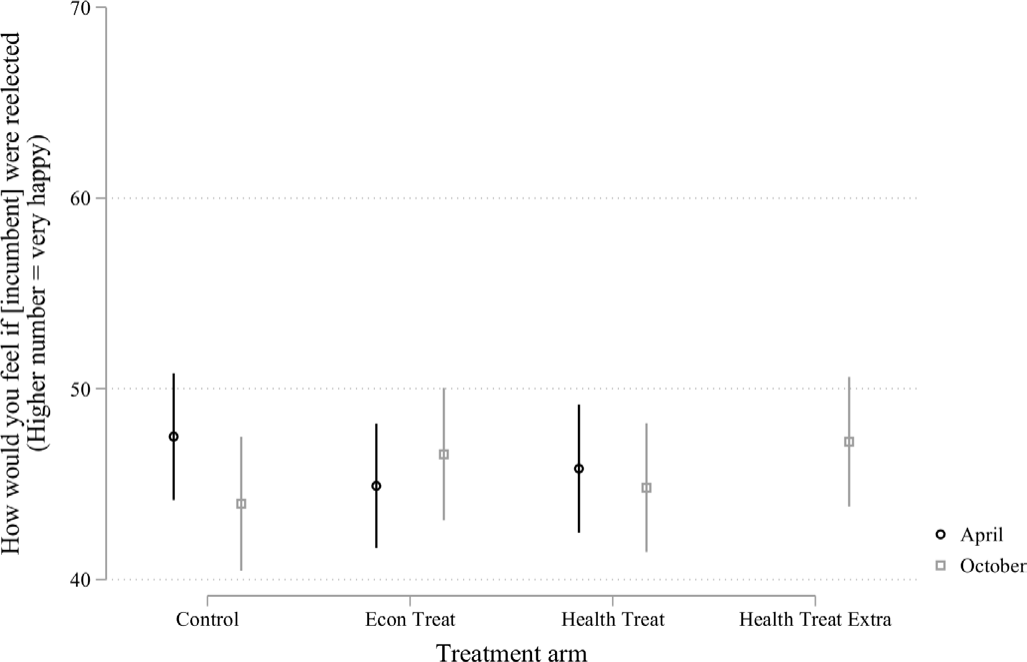
Incumbent support. Figure contains data from the USA collected in April and in October. The April data were discussed in our earlier paper.^1^ Vertical lines are 95% CIs.

More important, none of the treatment conditions—including the newly added arm, described above—have any impact on holding the government at fault (figure 1) or support for the incumbent (figure 2), replicating our earlier results.

Possible interpretations of this null result are discussed at length in the original paper. Here, we deal with these issues in the context of the US presidential election.

One interpretation of the null result is that public opinion in the USA is so partisan that nothing can move the needle, at least with respect to support for a sitting president.^3–5^ Yet, when we examine treatment effects among those whose political views lie closer to the middle of the spectrum—and hence should presumably be more susceptible to new information—we still observe null effects, as shown in the original paper. And the other countries in our initial survey—with, arguably, somewhat less polarised political environments—also show null effects. Moreover, other experimental studies of partisan or incumbent support in the USA (focused on non health-related treatments) often report significant (positive or negative) treatment effects,^6 7^ suggesting that null results are not ubiquitous. More generally, we note that the common complaint about experiments is that they are geared to achieve significant results. This is due, among other things, to respondents’ eagerness to provide whatever they perceive the researcher wishes to find.^8^

Another interpretation is that COVID-19 has now so thoroughly saturated people’s consciousness that no piece of information is informative. Yet, our initial survey was conducted at an earlier point in the progress of the virus, a point when one might imagine the level of awareness was lower.

The results of this survey experiment, and its predecessor, do not allow us to conclude that the pandemic had no impact on President Trump’s defeat. However, it does engender scepticism about the familiar narrative of a president held hostage by a virus. Note also that presidential approval ratings held remarkably steady over the past 2 years.^9^ The arrival of COVID-19 as a matter of public awareness early in 2020 does not register at all on this flat line. Of course, some may view Biden’s victory as evidence that Trump’s performance during the pandemic affected the outcome.^10^ It is difficult to say whether, or to what extent, the existence of the pandemic affected the US presidential election. Some voters blamed Trump for his lack of leadership, but they may have voted for Biden anyway, an interpretation supported by the fact that Biden led Trump in almost all major policy areas on the eve of the election.^11^ Others supported Trump’s position that the economy should be prioritised over the fight against COVID-19, but they may have already been strongly committed to the President. Our research suggests that COVID-19 changed few votes. However, there are many other ways in which this question can be addressed and we expect an outpouring of work to appear in the coming years.

What we can conclude is that this follow-up study reinforces doubts raised by our earlier study about the political relevance of pandemics. While previous survey experiments have found a whole host of treatments—centred on race, economics and other factors —move the needle for and against incumbents, we have not yet found a treatment associated with health that achieves the same results. More work is needed on these topics, especially because the political salience of pandemics is rather different from other health problems. In the interim, our findings suggest that the accountability link between citizens and politicians in the area of public health is weaker than we might have thought, or might like to believe.

## Data Availability

Data are available in a public, open repository - https://github.com/asreeves/health-voting-covid
